# Effectiveness and safety of acupuncture for elderly overactive bladder population in Hong Kong: study protocol for a randomized controlled trial

**DOI:** 10.1186/s13063-018-2706-4

**Published:** 2018-07-13

**Authors:** Yu Tat Chan, Hong Wei Zhang, Yuan Qi Guo, Tony Ngai Ho Chan, Yiu-Keung Kwan, Chun-Kam Lee, Kit Ngan, Zhi-Xiu Lin

**Affiliations:** 1 School of Chinese Medicine, Faculty of Medicine, The Chinese University of Hong Kong, Shatin, New Territories, Hong Kong, China; 2 Pok Oi Hospital - The Chinese University of Hong Kong Chinese Medicine Centre for Training & Research (Shatin), New Territories, Hong Kong, China; 3 Department of Medicine & Geriatrics, Tuen Mun Hospital, Tuen Mun, Hong Kong, China; 4 Hong Kong Institute of Integrative Medicine, Faculty of Medicine, The Chinese University of Hong Kong, Shatin, New Territories, Hong Kong, China

**Keywords:** Acupuncture, Elderly, Nerve growth factor, Overactive bladder, Sham controlled

## Abstract

**Background:**

Overactive bladder (OAB) is defined as “urgency, with or without urge incontinence, usually with frequency and nocturia”. Acupuncture is one of the most popular alternative treatment methods for OAB. Little established evidence is available to support the effectiveness of acupuncture for OAB. This study is a pioneer randomized, double-blinded, sham-controlled trial to assess the effectiveness and safety of acupuncture in the elderly population with overactive bladder in Hong Kong.

**Methods/design:**

This is a randomized, double-center, patient and outcome assessor blinded, sham-controlled trial. The study sample size is 100 patients. Eligible subjects aged between 60 to 90 years old will be recruited into this study. All subjects will be randomly allocated into the active acupuncture group or sham acupuncture group in a 1: 1 ratio. Participants who are allocated into the active acupuncture group will receive a standardized 30-min real acupuncture treatment session for a total of 16 sessions on the top of standard routine care, whilst those who are randomized to the sham acupuncture arm will receive sham acupuncture in addition to standard routine care. Non-penetrating needles will be utilized as sham acupuncture. The primary outcome measure is the 7-day voiding diary and the secondary outcome measures are urine nerve growth factor (NGF) level, the Incontinence Impact Questionnaire (IIQ-7), Urogenital Distress Inventory (UDI-6) and OAB Symptom Score (OABSS). All outcome measures will be collected at baseline, the end of treatment and 3 months after treatment completion.

**Discussion:**

The objectives of this study include (1) to evaluate the effectiveness and safety of acupuncture treatment in patients with OAB on reduction in the frequency of incontinence episodes as derived from a 7-day voiding diary, (2) to evaluate whether acupuncture treatment could improve subjective symptoms in patients with OAB and (3) to examine the feasibility of using NGF as a biomarker for overactive bladder and test correlation with the effectiveness of acupuncture intervention. The finding of this study will provide preliminary evidence on the effectiveness and safety of acupuncture for treatment of OAB.

**Trial registration:**

Chinese Clinical Trial Registry, ChiCTR-INR-16010048. Registered on 29 Nov 2016.

**Electronic supplementary material:**

The online version of this article (10.1186/s13063-018-2706-4) contains supplementary material, which is available to authorized users.

## Background

Overactive bladder, also known as OAB, or overactive bladder syndrome, is defined as “urgency, with or without urge incontinence, usually with frequency and nocturia” by the International Continence Society (ICS) [1]. OAB is usually classified into two different types, i.e. OAB dry and OAB wet type according to the clinical symptoms. OAB dry is defined as having ≧ 4 episodes of urgency in the previous 4 weeks with either frequency > 8 times per day or the use of ≧ 1 coping behavior to control bladder function. OAB wet meets all the criteria of the OAB dry, but with the addition of ≧ 3 episodes of incontinence in the past 4 weeks that are clearly not caused by stress incontinence [1].

A study by Emmons and Otto reported that around 17% of Americans suffered from OAB [2]. Stewart et al. demonstrated that the prevalence of OAB with and without urge incontinence in patients in the USA is age-specific, especially in the female population [3]. OAB with urge incontinence in women at 65–74 years of age was nine times as much as that in women at age 18–24 years [3]. On the other hand, a steady increase in the prevalence of OAB with advancing age has also been observed in the male population. According to “World Population Aging 2015”, in Hong Kong the population aged 60 years or more reached 21.7% of the total population by 2015 [4]. With the pace of population aging gathering speed, it is expected that there will be more patients with OAB in Hong Kong in the coming decades. In a recent study in Hong Kong, around 15% of the population was found to suffer from OAB [5]. The economic burden resulting from managing OAB in Hong Kong will be huge in the foreseeable future. Moreover, OAB can also cause severe social impact and lead to impaired quality of life for those sufferers [6].

The current treatment methods for OAB include pharmacological therapy, behavioral therapy and physical therapy. Unfortunately, the outcome of these methods is unsatisfactory. Medication is one of the most commonly used treatment methods in Hong Kong. Presently, anticholinergics remain the mainstay therapeutic drugs for OAB; however, these are only partially effective [2]. Moreover, these drugs have considerable side effects such as dry mouth, dry eye, cessation of perspiration or even cause photophobia or confusion. On the other hand, although a number of studies have reported that behavioral therapy and physical therapy are effective, their treatment effects are usually not sustainable, and usually decline 3 months after treatment [2].

Worldwide, acupuncture is perhaps the most popular alternative treatment method in OAB. Indeed, neurogenic bladder dysfunction is one of the indications for acupuncture recommended by the World Health Organization (WHO) [7]. Today, acupuncture is one of most widely practiced alternative and complementary treatment modalities in the Asia-Pacific region. Moreover, some clinical trials have been conducted to evaluate the effectiveness and the safety of acupuncture for the treatment of OAB. A pilot study conducted by Engberg et al. suggested that acupuncture may have a clinical meaningful effect on urge incontinence [8]. A clinical case report (*n* = 11) by Kitakoji et al. illustrated that acupuncture may elicit an improvement in OAB symptoms [9]. Furthermore, a comprehensive systematic review by the Agency for Healthcare Research and Quality of the U.S. Department of Health and Human Services on the treatment of OAB in women concluded that acupuncture produced intriguing results in relation to a decreased frequency of voiding and reduced symptoms of urgency, which were associated with changes in cystometrics [10]. These previous clinical trials and systematic review indicate that acupuncture has a potentially clinically meaningful treatment effect on OAB.

Nevertheless, several limitations of these studies may potentially bias the results and render the findings inconclusive. First, many studies were open-label trials or single blinded, i.e. with blinding not applied to patients or outcome assessors [2, 11, 12]. Second, some of the studies did not use a control arm. Third, the sample size of many trials was generally small [2, 12]. Fourth, many studies used the technique of sham acupuncture involving the penetration of skin on the non-specific acupuncture points, which the authors believed would not produce treatment effect [2]. However, it is well-known that any acupuncture involving penetration of the skin, regardless of whether or not the acupuncture points are specific, can elicit a physiologic response, hence a treatment effect [13]. Fifth, most of these studies focused only on female sufferers, and few evaluated the efficacy of acupuncture in male OAB sufferers [2, 8, 12, 14]. Last, the lack of objective outcome assessment for the outcome measurement is a major drawback of the previous studies of acupuncture for treatment of OAB.

Until now, there has been no appropriately designed clinical study to investigate the effectiveness and safety of traditional acupuncture treatment for OAB. There is a clear need to fill this knowledge gap. Based on the aforementioned points, a full-scale double-blind randomized controlled trial is urgently needed to establish evidence on the effectiveness and safety of acupuncture for treating OAB.

### Objectives

The primary objective of this proposed study is to evaluate the effectiveness of acupuncture treatment in patients with OAB on reduction in the frequency of incontinence episodes as recorded in a 7-day voiding diary.

The secondary objectives are to evaluate the safety of acupuncture treatment, determine whether acupuncture treatment could improve the subjective symptoms in patients with OAB, as well as to examine the feasibility of using nerve growth factor (NGF) as a biomarker for overactive bladder and to test correlation between this and the effectiveness of the intervention.

### Strengths and limitations of this study

The strengths of the study are as follows:

- This is a pioneer study to evaluate the effectiveness of acupuncture for treatment of overactive bladder using an objective outcome measure viz. urinary NGF, and to test correlation between urinary NGF and subjective symptoms.
- Most of the previous studies focused on female sufferers, whereas the proposed study includes both male and female patients. More solid evidence could be established on the effectiveness of acupuncture in treating OAB.
- A non-penetrative sham acupuncture device will be utilized in this study to get rid of an unexpected physiologic response
- Randomization is used to balance patients across two treatment groups.

The limitation of the study is that owing to the nature of acupuncture, it is not feasible to blind the acupuncturists who conduct the treatment procedure.

## Methods/design

### Study design

This is a double-center, patient and outcome assessor blinded, randomized, sham controlled trial to evaluate the effectiveness and safety of acupuncture treatment in patients with OAB, in Hong Kong. All eligible subjects (*n* = 100) will be randomly allocated to the intervention group (*n* = 50) or control group (*n* = 50) in a 1: 1 ratio. All subjects, both in the intervention and control group, would receive standard routine care. In the Hong Kong hospital setting, behavioral therapy and physical therapy are usually the standard routine care for overactive bladder. Acupuncture treatment will be either conducted in the Yan Oi Tong - The Chinese University of Hong Kong Chinese Medicine Centre for Training and Research in Tuen Mun (YOT-CUHKCMCTR) or The Chinese University of Hong Kong Chinese Medicine Specialty Clinic cum Clinical Teaching and Research Centre (CUHK-CMSCCTRC). This project is approved by the New Territories West Cluster Clinical and Research Ethics Committee of Hong Kong (NTWC/CREC/15147) and CUHK-New Territories East Cluster Clinical and Research Ethics Committee of Hong Kong (NTEC/CREC/2017.199-T). Written informed consent is obtained from each participant. The study will be undertaken in compliance with the principles of the International Conference on Harmonization (ICH) Good Clinical Practice (GCP) guidelines and the Declaration of Helsinki and is expected to be accomplished within a period of 24 months (Additional file 1).

### Participants

Patients who are diagnosed as having OAB and fulfill all the inclusion criteria and meet none of exclusion criteria (listed below) will be eligible to participate in the study. The diagnosis of the patients will be confirmed by two co-authors (TNHC and YKK) who are specialists in geriatrics.

### Subject recruitment

Patients will be recruited from the Geriatric Day Hospital and the Urology Specialist Clinic at Tuen Mun Hospital, CUHK-CMSCCTRC, YOT-CUHKCMCTR and other affiliated Chinese medicine clinics under the management of Yan Oi Tong.

As a back-up plan, we will also aim to recruit suitable patients from the community and the elderly centers in Hong Kong through advertisements. Moreover, our research team will approach Hong Kong OAB Associations to facilitate the recruitment process. Furthermore, we will also hold promotional talks in the community and write articles in the health column of local newspapers to boost subject recruitment if the number of subjects is not sufficient.

For the logistics of subject recruitment, a research assistant will go to the Geriatric Day Hospital and the Urology Specialist Clinic at Tuen Mun Hospital twice a week to identify and invite potentially eligible patients to participate in the study. The patients will be informed of the details of the study such as the objectives, scope, procedure and potential benefits and risks. The patient will be given a written informed consent form. Patients or their caretakers will be informed of all the details and potential risks of the study, and the written informed consent form will be signed and witnessed by the research assistant. Oral informed consent may be acceptable if the participants cannot read.

### Inclusion criteria

Patients will be eligible for inclusion if they are:

1. Male or female and are between 60 and 90 years old;
2. Suffering from OAB wet type with urge-associated incontinence at least twice during a 3-day period;
3. Physically and mentally able to complete the 7-day voiding diary, Urinary Distress Inventory (UDI-6), Overactive Bladder Symptom Score (OABSS) and Incontinence Impact questionnaire (IIQ7); and
4. Able to give written informed consent.

### Exclusion criteria

Patients will be excluded based on the following:

1. OAB symptoms caused by stroke or spinal injury;
2. Life threatening infection;
3. Unconsciousness or severe cognitive deficits;
4. Dementia caused by Alzheimer’s disease or other neurodegenerative diseases;
5. Previous incontinence surgery;
6. On short-term active diuretic treatment or taking diuretic medication;
7. Previous acupuncture treatment for OAB;
8. Current drug treatment for OAB;
9. Diagnosis of stress urinary incontinence;
10. Suffering from untreated urinary tract infection, urogenital tumors, prostate tumor, benign prostatic hyperplasia or chronic urinary retention; and
11. Immobility problems.

### Withdrawal criteria

The withdrawal criteria will be:

1. Participant’s decision to drop out from study at any time for any reason;
2. Non-adherence of participants to the treatment protocol - patients who complete < 80% of the treatment session; and
3. Occurrence of any unexpected serious side effects.

### Randomization and allocation concealment

**Fig. 1:**
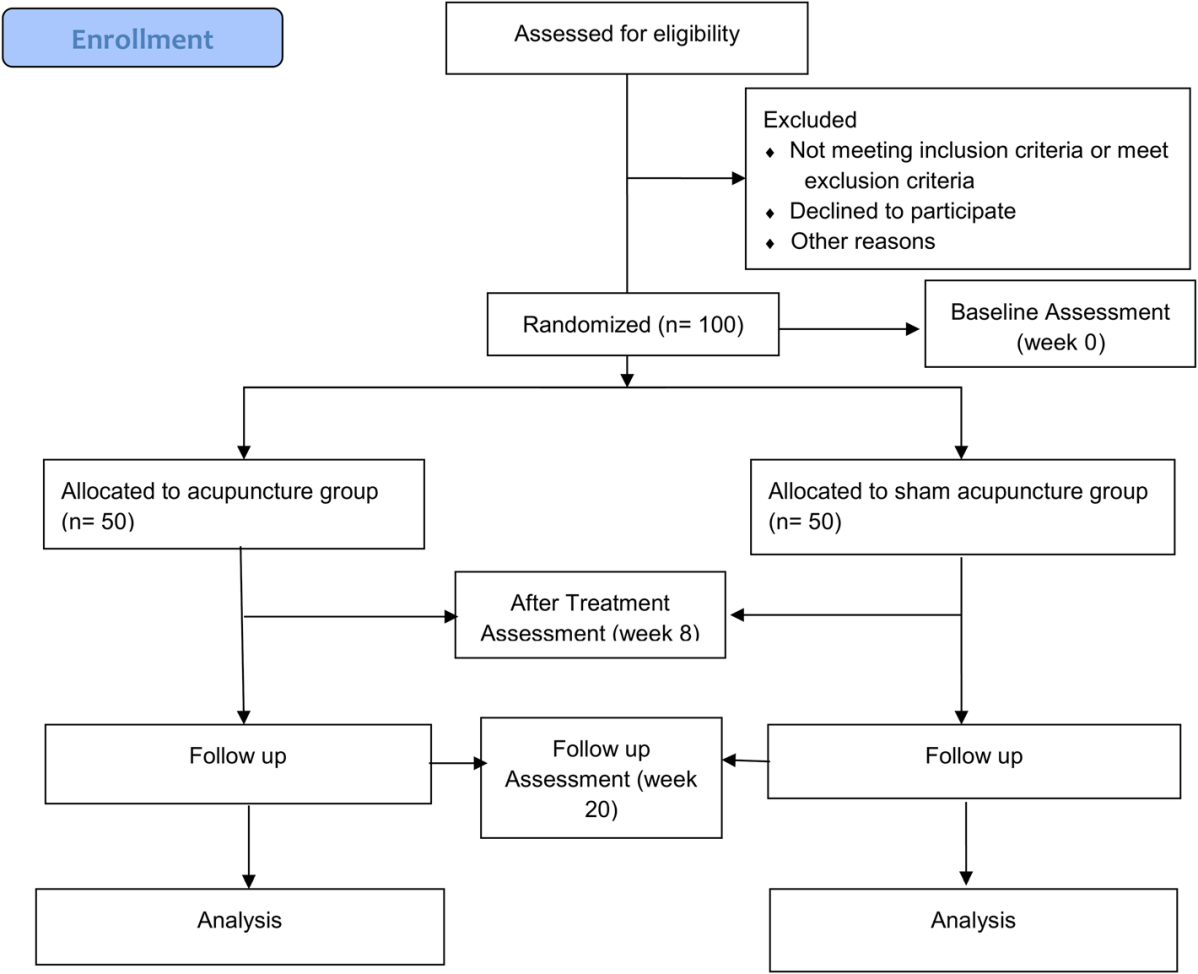
Study flow chart

The included participants will be randomly allocated to either the active acupuncture group (intervention group) or sham acupuncture group (placebo-control group) after the baseline assessment. We will use a computer program (Random Allocation Software, version 1.0; Msaghaei) [15] to generate random numbers. Block randomization will be used to ensure a balance in the number between the two groups. The list of randomization numbers will be kept by research staff who will be responsible for assigning the recruited patients to the corresponding intervention code based on the list. He or she will not be involved in patient care, outcome assessment, data collection or data analysis. The study flowchart is shown in Fig. 1.

### Blinding

All the participants, outcome assessors and clinical investigators will be kept blind to treatment allocation during the whole study. Owing to the nature of acupuncture, it is not feasible to blind the acupuncturists who perform the treatment procedure. However, they will be instructed to keep interaction with the patients to a minimum during the treatment period. Except for emergency cases, the blinding code will not be opened during the study period. To test the success of masking, a direct question will be asked to the participants: “Before the study was conducted, you were informed that you have equal chance to receive real acupuncture treatment and placebo-sham acupuncture treatment. After that, which treatment do you think you have received?”

### Interventions

Participants randomized to the intervention group will receive a standardized 30-min acupuncture treatment session in addition to standard routine care. The definition of standard routine care of overactive bladder in the Hong Kong hospital setting is all the conventional treatments except for medication, such as behavioral and physical therapy. From the Chinese medicine perspective, the pathogenesis of overactive bladder symptoms is mainly attributed to insecurity of kidney qi [16]. Pursuant to the traditional acupuncture theory, previous relevant studies and the opinion of acupuncture experts of the research team at the School of Chinese Medicine, CUHK, the following acupuncture points will be selected: BL32 (Ciliao) (bilateral), BL23 (Shenshu) (bilateral), SP6 (Sanyinjiao) (bilateral), KI3 (Taixi) (bilateral), BL39 (Weiyang) (bilateral), BL28 (Pangguangshu) (bilateral) and CV4 (Guanyuan).

All acupuncture treatment sessions will be performed by two acupuncturists who are registered Chinese Medicine Practitioners in Hong Kong and have at least 3 years clinical experience in acupuncture practice. Interventions will be conducted according to Standards for Reporting Interventions in Controlled Trials of Acupuncture (STRICTA) and ICH-GCP guidelines. To ensure the consistency of skill of various acupuncturists, several training workshops will be provided to acupuncturists who are involved in the study prior to the commencement of the study.

#### Active acupuncture group

Sterile disposable stainless steel needles of various lengths and diameters (Huatuo, Suzhou, China; 0.3 mm × 25 mm/0.3 mm × 40 mm/0.3 mm × 75 mm) will be used in the intervention group. The acupuncture treatment will be administered twice per week, which mimics the clinical practice of using acupuncture treatment for OAB in many Chinese medicine clinics in Hong Kong, and the whole treatment period will last for 8 consecutive weeks. A total of 16 sessions of acupuncture treatment will be administered to each participant.

#### Sham acupuncture group

In this study, participants randomized to the placebo-control arm after baseline assessment will receive sham acupuncture in addition to standard routine care. The sham acupuncture used in this study is a type of acupuncture in which needles are of the same size as those used in the intervention group; however, the needles will not penetrate the skin; instead, the needles are retracted into the needle handle instead of penetrating the skin and are directed at the same points as are used in the active treatment group. As such, subjects will experience a non-penetrating pricking sensation instead of *de-qi* feeling, characterized as soreness, numbness, heaviness and feeling of pressure.

**Fig. 2:**
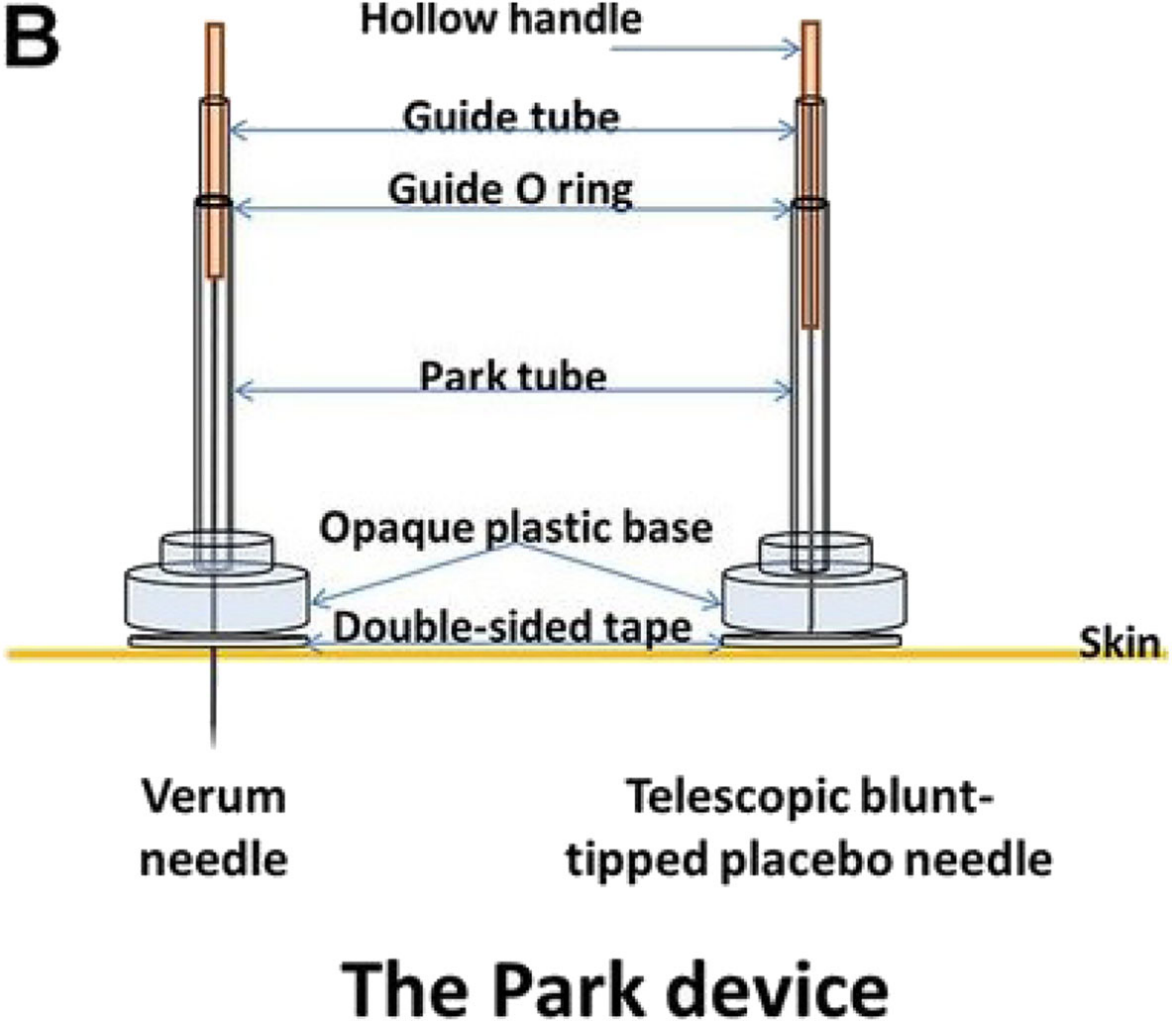
Structure of the Park Sham Device. Park Sham Device with sham needle and real needle. 1. Needle handle, 2. Guide tube, 3. Guide O-Ring, 4. Park tube, 5. Flange, 6. Double sided tape, 7. Skin 8. Dermis, 9. Muscle, 10. Dull tip of sham needle, 11. Sharp tip of real needle

The sham acupuncture device we will use is known as Park Sham Device (Dong Bang Acupuncture Inc., Korea), which consists of a base with a hole in the center and sticky tape on the bottom. The sham acupuncture device is specifically designed for sham-acupuncture-controlled trials, and makes it impossible for the subject to see whether or not the needle has penetrated the skin. Each Park Sham Device is composed of a needle and a series of components, for instance, an adhesive tube, a guide tube, the Park tube and a flange [17]. The structure of the Park Sham Device is shown in Fig. 2.

As an incentive measure for patient compliance and for ethical considerations, for patients who are assigned to the sham acupuncture group, we will provide free acupuncture treatment for their OAB after they have completed the entire study including the follow-up period (Table 1).

**Table 1:** Locations, therapeutic indications and manipulations of the acupuncture points selected in this study

Points	Location	Indications	Manipulation
BL32 (次髎)	On the sacrum, medial and inferior to the posterosuperior iliac spine, just at the 2nd posterior sacral foramen	Lumbago, hernia, irregular menstruation, abnormal discharge, dysmenorrheal, emission, impotence, enuresis, dysuria, flaccidity of lower extremities	Puncture perpendicularly to a depth of 1.2 cun, then apply reinforcing technique with the lifting and thrusting movement for 30 s
BL23 (腎俞)	On the lower back, below the spinous process of the 2nd lumbar vertebra, 1.5 cun lateral to the posterior midline	Emission, impotence, enuresis, irregular menstruation, leukorrhagia, lumbago, soreness and weakness of the waist and knee, dizziness and vertigo, tinnitus, deafness, edema, dyspnea, diarrhea	Puncture perpendicularly to a depth of 1.2 cun, then apply reinforcing technique with the lifting and thrusting movement for 30 s
SP6 (三陰交)	On the medial side of the leg,3 cun above the tip of the medial malleolus, posterior to the medial border of the tibia	Abdominal pain, borborygmus, distention of the abdomen, diarrhea, dysmenorrhea, irregular menstruation, metrostaxis and metrorrhagia, abnormal vaginal discharge, prolapse of the uterus, infertility, dystocia, emission, impotency, enuresis, dysuria, edema, hernia, pain in the vulva, flaccidity of the lower extremities, headache, dizziness, insomnia	Puncture perpendicularly to a depth of 1.0 cun, then apply reinforcing technique with the lifting and thrusting movement for 30 s
KI3 (太溪)	On the medial border of the foot, posterior to the medial malleolus, in the depression between the tip of the medial malleolus and Achilles tendon	Dry and sore throat, toothache, deafness, tinnitus, dizziness, hemoptysis, dyspnea, diabetes mellitus, irregular menstruation, insomnia, emission, impotence, frequency of micturition, lumbago	Puncture perpendicularly to a depth of 0.8 cun, then apply reinforcing technique with the lifting and thrusting movement for 30 s
BL39 (委陽)	At the lateral end of the popliteal crease, medial to the tendon of the biceps muscle of the thigh	Stiffness of the back and lumbar region, distention of the lower abdomen, edema, dysuria, contraction of the leg and foot.	Puncture perpendicularly to a depth of 1.0 cun, then apply reinforcing technique with the lifting and thrusting movement for 30 s
BL28 (膀胱俞)	On the sacrum and on the level of the 2nd posterior sacral foramen, 1.5 cun lateral to the median sacral crest.	Interrupted urination, enuresis, frequency of micturition, diarrhea, constipation, stiffness and pain in the back and lumbar region	Puncture perpendicularly to a depth of 1.0 cun, then apply reinforcing technique with the lifting and thrusting movement for 30 s
CV4 (關元)	On the lower abdomen and on the anterior midline, 3 cun below the center of the umbilicus	Enuresis, nocturnal emission, frequency of micturition, retention of urination, hernia, irregular menstruation, abnormal vaginal discharge, metrorrhagia and metrostaxis, postpartum hemorrhage. Pain in the lower abdomen, indigestion, diarrhea, rectal prolapse, apoplexy marked by prostration syndrome	Puncture perpendicularly to a depth of 1.0 cun, then apply reinforcing technique with the lifting and thrusting movement for 30 s

### Outcomes

#### Primary outcome

Among these variables, the primary outcome measurement is change in the frequency of incontinence episodes [2]. The 7-day voiding diary, which records daily micturition and symptoms, such as the time of micturition, voided volume per micturition and incontinence episodes in the most recent 7 days. The 7-day voiding diary is chosen based on the following reasons: (a) the validity of the scale has been proven by a previous study, (b) it was found to provide more accurate information than the 3-day voiding diary [18], (c) it is a non-invasive assessment tool, which minimizes the risk and inconvenience to the patient and (d) the Chinese version of the diary has been validated and is readily available for use.

#### Secondary outcomes

##### Urinary NGF

Lack of objective outcome assessment is a major limitation of the previous studies on OAB. Aydogus et al. [19] conducted a pioneer placebo-controlled clinical trial of acupuncture for treating OAB using NGF as a biological marker for treatment outcome. It was reported that the decrease in urinary NGF caused by acupuncture and solifenacin treatment correlated well with the improvement in OAB symptoms. Based on these findings, we propose to use urinary NGF as a secondary outcome in the present study. The urine sample will be collected from each participant at baseline and weeks 8 and 20 (follow up) and the urinary NGF will be measured by ELISA. The laboratories at the School of Chinese Medicine, CUHK have all the necessary facilities to conduct the ELISA [20] (Table 2).

**Table 2:** Summary of outcome measurements at different time points

Outcome measurements	Data collection instrument	Baseline	Treatment	Follow up
			Week 8 (end of treatment)	Week 20 (end of follow-up period)
The 7-day voiding diary	Diary	✓	✓	✓
Urinary NGF level	ELISA	✓	✓	✓
IIQ-7	Questionnaire	✓	✓	✓
UDI-6	Questionnaire	✓	✓	✓
Adverse events	Adverse event report form		✓	

*NGF* nerve growth factor, *IIQ-7* Incontinence Impact Questionnaire, *UDI-6* Urogenital Distress Inventory

##### Incontinence Impact Questionnaire (IIQ-7), Overactive Bladder Symptom Score (OABSS) and Urogenital Distress Inventory (UDI-6)

Besides urinary NGF, the outcomes recommended by the ICS, such as IIQ-7 and UDI-6 will also be used as secondary outcome measures [21]. The IIQ-7 and UDI-6 chosen for this study are available in Chinese versions, which are known to be as sensitive, valid and reliable as the original version and other languages [22]. Moreover, a simple self-report questionnaire consisting of four symptoms - daytime frequency, night-time frequency, urgency and urgency incontinence will also be used as a secondary outcome [23].

**Fig. 3:**
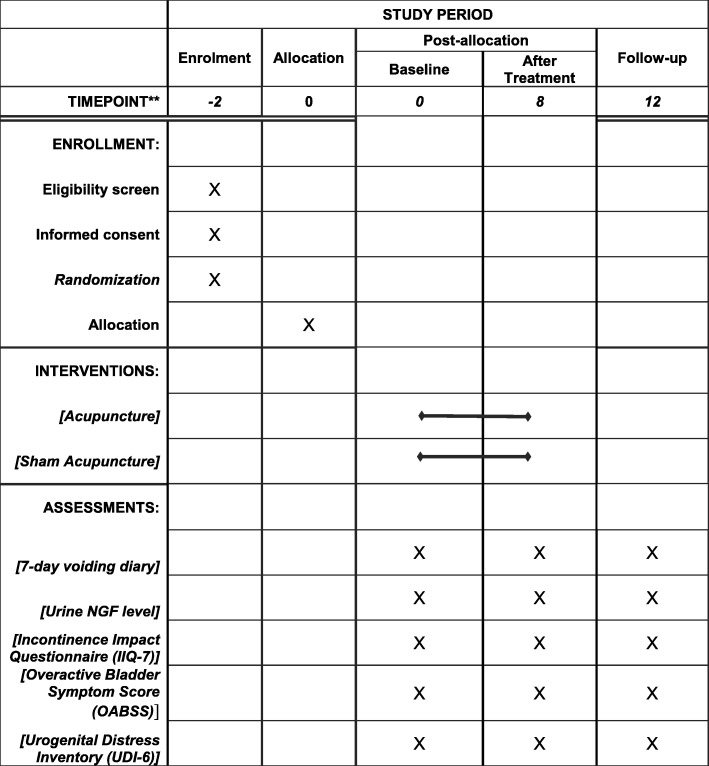
Schedule of enrollment, intervention and assessments of this study protocol

All of the primary and secondary outcomes will be collected at baseline, the end of treatment (2 months after inclusion) and 3 months after the completion of treatment. The schedule of enrolment, intervention and assessments is shown in Fig. 3.

### Adverse events

All adverse events as defined by ICH-GCP guidelines will be fully recorded on the adverse event pages of the case report forms (CRFs). Signs and symptoms of each adverse event will be described in detail such as date of onset, intensity, outcome, date of resolution and any action taken. The adverse events will be tabulated in each group and the incidences calculated. Serious adverse events will be reported to the Research Ethics Committee concerned within 24 h. If any serious adverse effect occurs, emergency medical assistance will be sought and all details noted. The adverse events are recorded for the purpose of evaluating the safety of acupuncture treatment.

### Sample size estimation

The primary outcome measure is post-treatment incontinence episodes as derived from the 7-day bladder diary. Based on a previous study of Emmons and Otto [2], the placebo effect was 40% reduction in incontinence episodes among patients treated with placebo acupuncture. To detect a clinically meaningful effect of 30% difference in the change of frequency of incontinence episodes between the treatment and placebo control group, 41 cases will be needed in each group to have 80% power to detect difference between the two groups at the significance level of 0.05. To allow for a predicted dropout rate of 20%, a total of 100 patients will be required for this study, with 50 patients in each arm.

### Data management and monitoring

The study will be conducted in the YOT-CUHKCMCTR and CUHK-CMSCCTRC in accordance with the research protocol, ICH GCP guidelines, the Declaration of Helsinki and the applicable laws of Hong Kong. The investigators will assure that (1) the rights and wellbeing of the participants are protected; (2) the reported trial data are accurate, complete and verifiable from source documents; (3) the information on the patients is secure and (4) the trial complies with the protocol and applicable regulatory requirements. The research assistant who has undertaken ICH GCP training will maintain complete participant records. Relevant data are then transcribed into the CRF. The CRFs will be completed for each participant enrolled in the study and will be signed by the Principal Investigator. This would be done as soon as possible after completion of a study visit. All data will be put onto the computer with password protection. The data will be double checked by investigators after data entry. In addition, we will comply with the Consolidated Standards of Reporting Trials (CONSORT) chart when reporting the results of this study.

### Statistical analysis

All analyses will be conducted according to the intention-to-treat principle. Descriptive statistics will be computed for each of the analyzed variables. The baseline characteristics will be tabulated and some of large differences between the two groups will be noted and examined in the general mixed model. The generalized linear mixed model will be used to compare primary and secondary outcomes between groups and test correlation between baseline factors, including NGF level, and the primary and secondary outcomes. Per-protocol analysis will be also performed. All statistical tests will be two-sided, and *p* < 0.05 is considered statistically significant. The statistical software of SPSS (SPSS, SPSS Inc., Chicago, USA) version 23.0 will be used for the analysis.

## Discussion

OAB is a common disease among the elderly in many countries. The economic expenditure in managing OAB is enormous according to the data from six industrialized countries, including Canada, Germany, Italy, Spain, Sweden and the UK [11].

Acupuncture has been widely used in clinical practice in many countries for the treatment of OAB. However, until now, there has been no appropriately designed randomized control trial to provide explicit evidence about the effectiveness of acupuncture for treatment of OAB worldwide. To evaluate whether acupuncture treatment is effective in the management of OAB, a full-scale randomized, double-blinded, sham-controlled trial of acupuncture is proposed.

A standardized treatment protocol will be utilized to assure reproducibility of the study. The acupuncture point selection of the treatment protocol was based on traditional acupuncture theory, previous studies and the consensus of the acupuncture expert panel at the School of Chinese Medicine, The Chinese University of Hong Kong.

Change in the frequency of incontinence episodes as obtained from 7-day voiding diaries was chosen as the primary outcome. The severity of OAB is mainly relied on patient-reported outcomes, and voiding diary is widely used as an outcome measure in OAB studies. It is safe and convenient, and also various language version diaries are readily available. Moreover, a previous study [18] demonstrated that the 7-day voiding diary provides more accurate information than the 3-day voiding diary.

One major limitation of previous studies of acupuncture for treatment of OAB is the lack of an objective outcome assessment, and this lack may weaken the creditability of the finding. The significant novelty of this proposed study is to introduce an objective biomarker as a secondary outcome. Stanley Cohen and Rita Levi-Montalcini, the Nobel Prize Winners in Physiology or Medicine in 1998, discovered NGF, dating back to 1950. NGF is a small protein that can induce differentiation and affect particular nerves specifically. In recent years, many studies have reported that NGF can be used as potential biomarker in OAB. The review conducted by Seth et al. [24] gives a comprehensive summary of all previous studies that examined urinary NGF. Indeed, it has been established that there is close correlation between symptoms of OAB and urinary NGF [25]. Based on the current understanding of NGF, a more objective outcome measure, viz. urinary NGF, has been proposed as a secondary outcome in our study. In addition, the IIQ-7, OAB Symptom Score and UDI-6 were selected as secondary outcome measures to evaluate the psychosocial impact and quality of life of patients with OAB. Those structural questionnaires have been validated in the Chinese patients with OAB.

This study is funded by the Health and Medical Research Fund of Hong Kong, and the study protocol describes the first full-scale randomized controlled trial evaluating the effectiveness and safety of acupuncture in patients with OAB. The findings of the study will provide evidence on the effectiveness and safety of acupuncture for OAB. Moreover, the results of this trial will form a foundation for further study to compare the effectiveness of acupuncture with the usual pharmacological treatment.

### Trial status

This trial is currently ongoing. The study commenced on 1September 2016, and the anticipated end date of the study is 31 August 2018.

## Additional file

Additional file 1: Completed Standard Protocol Items: Recommendation for Interventional Trials (SPIRIT) 2013 Checklist: items addressed in this clinical trial protocol and related documents [26]. (DOCX 61 kb)

## Abbreviations

CRF: Case report form
CUHK-CMSCCTRC: The Chinese University of Hong Kong Chinese Medicine Specialty Clinic Clinical Teaching and Research Centre
ELISA: Enzyme-linked immunosorbent assay
GCP: Good Clinical Practice
ICH: International Conference on Harmonization
ICS: International Continence Society
IIQ-7: Incontinence Impact Questionnaire
NGF: Nerve growth factor
OAB: Overactive bladder
UDI-6: Urogenital Distress Inventory
YOT-CUHKCMCTR: Yan Oi Tong - The Chinese University of Hong Kong Chinese Medicine Centre for Training and Research in Tuen Mun
